# Diagnostic values of serum BNP, PTX3, and VEGF in acute pulmonary embolism complicated by pulmonary artery hypertension and their correlations with severity of pulmonary artery hypertension

**DOI:** 10.1002/iid3.986

**Published:** 2023-09-20

**Authors:** Qinghou Zheng, Bin Zhang, Na Lu, Xinyi Li, Beibei Jin, Pingkui Jin

**Affiliations:** ^1^ Department of Emergency The First Hospital of Hebei Medical University Shijiazhuang Hebei China

**Keywords:** acute pulmonary embolism, BNP, diagnostic value, disease severity, PTX3, pulmonary artery hypertension, VEGF

## Abstract

**Objective:**

This paper aimed to unveil the diagnostic values of serum brain natriuretic peptide (BNP), pentraxin 3 (PTX3), and vascular endothelial growth factor (VEGF) in acute pulmonary embolism complicated by pulmonary artery hypertension (APE‐PAH) and their correlations with severity of PAH.

**Methods:**

A total of 153 patients with APE were selected for our study and divided into the PAH and Non‐PAH groups according to the measurement of pulmonary artery pressure by echocardiography. Serum BNP levels were measured by chemiluminescence immunoassay, and serum PTX3 and VEGF levels were appraised by ELISA. The predictive values of BNP, PTX3, and VEGF for APE‐PAH were evaluated by applying the receiver operating characteristic (ROC) curve. Spearman test was implemented to correlate BNP, PTX3, and VEGF with the severity of PAH.

**Results:**

Higher serum levels of BNP, PTX3, and VEGF were observed in the PAH group versus the Non‐PAH group (*p* < .05). ROC curve analysis indicated that BNP, PTX3, and VEGF had acceptable diagnostic value for predicting APE‐PAH. Higher serum levels of BNP, PTX3, and VEGF were witnessed in the moderate and severe PAH groups in contrast to the mild PAH group (*p* < .05), and the levels of these parameters were elevated in the severe PAH group versus the moderate PAH group (*p* < .05). Spearman correlation analysis signified that serum BNP (*r* = 0.377), PTX3 (*r* = 0.488), and VEGF (*r* = 0.575) levels were positively correlated with the severity of PAH in APE‐PAH patients.

**Conclusion:**

Serum BNP, PTX3, and VEGF levels are significantly elevated in APE‐PAH patients. Serum BNP, PTX3, and VEGF levels are of clinical value in the diagnosis of APE‐PAH patients, and serum BNP, PTX3, and VEGF levels are positively correlated with the severity of PAH and can be used as predictors of the severity of PAH.

## Graphical abstract

In summary, our paper signifies that serum brain natriuretic peptide, pentraxin 3, and vascular endothelial growth factor are of clinical values as biological indicators for predicting acute pulmonary embolism complicated by pulmonary artery hypertension (APE‐PAH) and reflecting the severity of APE‐PAH. Further investigations are necessary to unveil their complex interactions of these factors in the development of APE‐PAH.

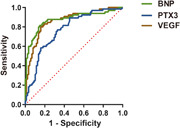

## 1 INTRODUCTION

Acute pulmonary embolism (APE), occurring after intrapulmonary changes, is a frequent and potentially fatal disease that results in a range of cardiopulmonary consequences and even sudden death.^1^ Available management options for APE consist of systemic thrombolysis, surgical embolectomy, catheter‐based thrombolysis or embolectomy, as well as extracorporeal membrane oxygenation support.^2^ Nevertheless, due to rapid development in surgical and nonsurgical approaches, an established therapy algorithm for APE remains to be established.^3^ APE is able to lead to pulmonary artery hypertension (PAH), induced by macroscopic or microscopic obstruction of the pulmonary vasculatures and active vasoconstriction, because of the released vasoconstrictive mediators and scavenging of nitric oxide by cell‐free hemoglobin.^4^ PAH is a multifactorial vascular disorder and caused by the interaction of chronic hemolysis, platelet activation, oxidative stress, dysfunctional angiogenesis, and iron overload.^5^ The correct and early increase of pulmonary arterial pressure (PAP) is crucial to decide the proper management and follow‐up for PAH.^6^ Great advances have been achieved in the treatment of PAH, but the underlying pathophysiological heterogeneity of PAH impairs treatment efficacy, contributing to elevated morbidity and mortality rate.^7^ Since PAH is a progressive, life‐threatening, and irreversible disorder, a simple and available tool for the estimation of the individualized risk and progression of PAH is urgently needed.

Brain natriuretic peptide (BNP) is a recently discovered peptide synthesized and secreted by the atria and ventricles and after digestion, it becomes active BNP and NTproBNP.^8^ BNP reduces blood volume through enhancing systemic vascular permeability and natriuretic actions, and eases vascular smooth muscle cells through enhancing intracellular cGMP synthesis, thereby decreasing blood pressure and ventricular preload. NTproBNP/BNP has been broadly utilized as noninvasive and prognostic hallmarks of cardiac dysfunction and the sole biomarker recommended by the risk stratification guidelines for PAH.^9^ Pentraxin 3 (PTX3) has a high‐affinity binding site with fibroblast growth factor 2 (FGF2) on its N‐terminal domain, which gives it unique suppressive properties against the FGF2‐mediated changes.^10^ Serum PTX3 levels correlate with the degree of infectious disease and serve as a prognostic hallmark in bacteriemia and fatal diseases.^11^, ^12^ Besides, PTX3 has also been recently revealed as a specific biomarker for PAH diagnosis.^13^ As reported, angiogenic and angiostatic factors, including vascular endothelial growth factor (VEGF), are implicated in PAH development.^14^ VEGF is a pivotal mediator of endothelial proliferation, angiogenesis, as well as vascular remodeling, which exerts functions in PAH development.^15^ Moreover, both in animal models and non‐hematological disorders, the role of VEGF in PAH development has been widely discussed.^16^, ^17^ Based on the aforesaid articles, this paper aimed to unveil the diagnostic values of serum BNP, PTX3, and VEGF in APE‐PAH and their correlations with the severity of PAH to provide a scientific basis for early diagnosis and intervention for patients.

## 2 MATERIALS AND METHODS

### 2.1 Participants

The 153 patients with APE admitted to our hospital were enrolled, and the basic information of the study subjects were detailed in Table 1.

**Table 1:** Comparison of general information between the two groups of patients.

	Non‐PAH group (*n* = 88)	PAH group (*n* = 65)	*p*
Age (years)	65.77 ± 6.98	67.20 ± 5.66	.179
Gender (male/female)	52/36	35/30	.621
Body mass index (kg/m^2^)	22.27 ± 1.83	23.02 ± 2.01	.425
Heart rate (time/min)	80.43 ± 14.55	84.58 ± 15.46	.949
Disease time (day)	4.64 ± 2.13	4.87 ± 2.11	.532
Systolic pressure (mmHg)	123.34 ± 20.6	124.29 ± 23.57	.742
Diastolic pressure (mmHg)	81.47 ± 11.09	77.56 ± 11.84	.120
PaO_2_ (mmHg)	73.58 ± 6.54	72.08 ± 6.59	.164
PaCO_2_ (mmHg)	37.31 ± 4.19	38.56 ± 4.38	.076

Abbreviation: PAH, pulmonary artery hypertension.

Patients were enrolled in this research if they met the following inclusion criteria: (1) CT pulmonary arteriogram: visualization of intra‐pulmonary artery thrombosis; immediate signs: filling defect in the pulmonary artery, partial or complete obstruction, orbital signs, no visualization of distal vessels; (2) ECT nuclide lung ventilation/perfusion scan (V/Q scan): lung perfusion defects distributed across lung segments and mismatched with lung ventilation phenomena (ventilation/perfusion mismatch in two or more lung segments, that is, normal ventilation scan in perfusion‐deficient lung segments); (3) echocardiography: proximal pulmonary artery thrombosis or right heart thrombosis; (4) the patient and family signed an informed consent form and underwent tests for PTX3, BNP, and VEGF.

Patients were excluded if they combined with other parts of embolism or previous history of pulmonary embolism; with acute or chronic inflammatory diseases, or respiratory diseases; with severe liver and kidney insufficiency, immune diseases, and malignant tumors; those who were allergic to contrast agents and could not perform pulmonary artery enhancement CT examination; those with acute cerebrovascular disease, acute coronary syndrome, and acute myocardial infarction; women during pregnancy and lactation.

### 2.2 Baseline data

Basic information was collected from all study subjects, including age, gender, height, weight, systolic blood pressure, diastolic blood pressure, heart rate, PaO_2_, and PaCO_2_.

### 2.3 Pulmonary artery systolic pressure (PASP) measurement and PAP grouping

All APE patients underwent echocardiography within 24 h after admission and were allocated into the PAH group (PASP ≥ 30 mmHg) and Non‐PAH group (PASP < 30 mmHg) following the PAP measured by echocardiography after admission. Patients in the PAH group were subdivided into the mild PAH group (PASP of 30−49 mmHg; moderate PAH group (PASP of 50−69 mmHg, and severe PAH group (PASP of 70 mmHg and above).

### 2.4 Detection of serum parameters

The fasting venous blood was drawn from all patients within 24 h of admission, centrifuged at 3000 r/min for 10 min, and the serum was separated. Serum BNP levels were determined using a chemiluminescence immunoassay kit (Roche Diagnostics). Serum PTX3 and VEGF levels were measured by enzyme‐linked immunosorbent assay (ELISA) kits (Boster).

### 2.5 Adverse cardiovascular events

Major adverse cardiovascular event (MACE, defined as cardiogenic death, right heart failure, and recurrent pulmonary thromboembolism) were observed and recorded during hospitalization and 1 month after discharge in both groups.

### 2.6 Statistical methods

The data were processed using SPSS20.0 statistical software (IBM). Numeration data were expressed as the number of cases, and the chi‐square test or Fisher's exact test was employed for comparison between groups. Measurement data were represented with mean ± standard deviation, and the *t*‐test was implemented for comparisons in two groups. The diagnostic values of BNP, PTX3, and VEGF in patients with APE‐PAH were analyzed using the receiver operating characteristic (ROC) curves, and correlation analysis was executed using the Spearman test. A *p* Value less than .05 meant a statistical significance.

## 3 RESULTS

### 3.1 General information comparison

In this paper, a total of 153 patients with APE were selected for our study and divided into the PAH and Non‐PAH groups according to the measurement of PAP by echocardiography. There were 52 males and 36 females in the Non‐PAH group, and patients aged 49−79 years with a mean age of (65.77 ± 6.98) years; there were 35 males and 30 females in the PAH group, and patients aged 56−77 years with a mean age of (67.20 ± 5.66) years. The differences in age, gender, body mass index, heart rate, disease time, systolic pressure, diastolic pressure, PaO_2_, and PaCO_2_ levels exhibited no statistical significance in the Non‐PAH and PAH groups (all *p* > .05), which were comparable (Table 1).

### 3.2 Echocardiography examination

The echocardiographic parameters (PASP, right ventricular internal dimension, left ventricle ejection fraction, and tricuspid regurgitation velocity) in patients in the Non‐PAH and PAH groups were compared, and the findings indicated that there exhibited elevated PASP, right ventricular internal dimension, left ventricle ejection fraction, and tricuspid regurgitation velocity in the PAH group in contrast to the Non‐PAH group (all *p* < .05) (Table 2).

**Table 2:** Comparison of echocardiography in patients of the two groups.

Parameter	Non‐PAH group (*n* = 88)	PAH group (*n* = 65)	*p*
Pulmonary arterial systolic pressure (mmHg)	23.11 ± 2.57	55.26 ± 10.52	<.001
Right ventricular internal dimension (mm)	41.55 ± 5.89	44.82 ± 5.27	<.001
Left ventricle ejection fraction (%)	59.44 ± 4.35	63.38 ± 4.49	<.001
Tricuspid regurgitation velocity (m/s)	1.66 ± 0.51	2.25 ± 0.81	<.001

Abbreviation: PAH, pulmonary artery hypertension.

### 3.3 Incidence of MACE

The incidence of MACE in the PAH group was 48/65 (73.85%), which was higher than that in the Non‐PAH group, 14/88 (15.91%) (*p* < .05; Table 3).

**Table 3:** MACE in patients of the two groups.

	Non‐PAH group (*n* = 88)	PAH group (*n* = 65)	*p*
MACE	14 (15.91%)	48 (73.85%)	<.001

Abbreviations: APE, acute pulmonary embolism; PAH, pulmonary artery hypertension; MACE, major adverse cardiovascular event.

### 3.4 Serum levels of BNP, PTX3, and VEGF in the Non‐PAH and PAH groups

Serum BNP, PTX3, and VEGF levels in subjects from the Non‐PAH and PAH groups were compared and the results disclosed that higher serum levels of BNP, PTX3, and VEGF were observed in the PAH group versus the Non‐PAH group (all *p* < .05) (Table 4).

**Table 4:** Serum levels of BNP, PTX3, and VEGF in patients of the two groups.

Parameter	Non‐PAH group (*n* = 88)	PAH group (*n* = 65)	*p*
BNP (pg/mL)	196.15 ± 20.38	240.42 ± 30.73	<.001
PTX3 (ng/mL)	5.62 ± 1.13	6.79 ± 1.22	<.001
VEGF (pg/mL)	303.33 ± 29.62	361.96 ± 31.85	<.001

Abbreviations: PAH, pulmonary artery hypertension; BNP, brain natriuretic peptide; PTX3, pentraxin 3; VEGF, vascular endothelial growth factor.

### 3.5 Analysis of the predictive values of BNP, PTX3, and VEGF for APE‐PAH

**Figure 1:**
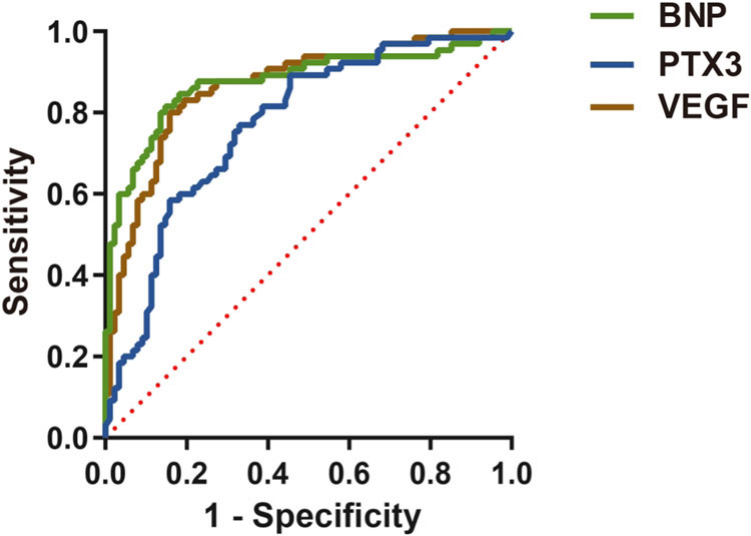
Analysis of the predictive values of BNP, PTX3, and VEGF in APE‐PAH patients. APE‐PAH, acute pulmonary embolism complicated by pulmonary artery hypertension; BNP, brain natriuretic peptide; PTX3, pentraxin 3; VEGF, vascular endothelial growth factor.

ROC curve was applied to analyze the predictive values of BNP, PTX3, and VEGF in patients with APE‐PAH, and the findings uncovered that BNP, PTX3, and VEGF had some diagnostic values in patients with APE‐PAH. The area under the curve (AUC) for prediction of APE‐PAH by BNP was 0.876 (95% confidence interval [CI]: 0.815−0.938, *p* < .001), with a sensitivity and specificity of 0.815 and 0.852 when the cutoff value was 216.22 pg/mL. The AUC for prediction of APE‐PAH by PTX3 was 0.771 (95% CI: 0.696−0.846, *p* < .001), with a sensitivity of 0.769 and specificity of 0.670 when the cut‐off value was 5.94 ng/mL. The AUC for prediction of APE‐PAH by VEGF was 0.864 (95% CI: 0.805−0.924, *p* < .001), with a sensitivity of 0.800 and specificity of 0.841 when the cut‐off value was 329.49 pg/mL (Table 5 and Figure 1).

**Table 5:** Analysis of the predictive values of BNP, PTX3, and VEGF for APE‐PAH.

Parameter	AUC	Cutoff value	Sensitivity	Specificity	95% CI, *p*
BNP	0.876	216.22 pg/mL	0.815	0.852	0.815−0.938, <.001
PTX3	0.771	5.94 ng/mL	0.769	0.670	0.696−0.846, <.001
VEGF	0.864	329.49 pg/mL	0.800	0.841	0.805−0.924, <.001

Abbreviations: APE, acute pulmonary embolism; AUC, area under the curve; BNP, brain natriuretic peptide; PAH, pulmonary artery hypertension; PTX3, pentraxin 3; VEGF, vascular endothelial growth factor.

### 3.6 Comparison of differences in BNP, PTX3, and VEGF levels between different PAH subgroups

Serum BNP, PTX3, and VEGF levels were compared between different PAH subgroups. The results unveiled that higher serum levels of BNP, PTX3, and VEGF were witnessed in the moderate and severe PAH groups in contrast to the mild PAH group, and the levels of these parameters were elevated in the severe PAH group versus the moderate PAH group (all *p* < .05) (Table 6).

**Table 6:** Comparison of BNP, PTX3, and VEGF levels in patients between different PAH subgroups.

Parameter	Mild PAH group (*n* = 20)	Moderate PAH group (*n* = 38)	Severe PAH group (*n* = 7)
BNP (pg/mL)	226.44 ± 36.00	242.83 ± 25.41*	267.26 ± 21.45* ^,^ **
PTX3 (ng/mL)	6.13 ± 1.08	6.72 ± 0.83*	9.03 ± 0.78* ^,^ **
VEGF (pg/mL)	331.66 ± 26.40	354.57 ± 28.06*	395.79 ± 9.99* ^,^ **

Abbreviations: BNP, brain natriuretic peptide; PAH, pulmonary artery hypertension; PTX3, pentraxin 3; VEGF, vascular endothelial growth factor.

* *p* < .05 versus the mild PAH group;

** *p* < .05 versus the moderate PAH group.

### 3.7 Correlations of BNP, PTX3, and VEGF with the severity of PAH

Spearman test was further implemented to analyze the correlations of serum BNP, PTX3, and VEGF levels and the severity of PAH, and the findings signified that that serum BNP, PTX3, and VEGF levels were positively correlated with the severity of PAH (mild, moderate, and mild) in APE‐PAH patients (*r* = 0.377, 0.488, 0.575, all *p* < .05; Table 7).

**Table 7:** Correlations of BNP, PTX3, and VEGF with severity of PAH.

Severity of PAH		
	*r*	*p*
BNP	0.377	.002
PTX3	0.488	<.001
VEGF	0.575	<.001

Abbreviations: BNP, brain natriuretic peptide; PAP, pulmonary artery pressure; PTX3, pentraxin 3; VEGF, vascular endothelial growth factor.

## 4 DISCUSSION

APE‐PAH stems from interactions of diverse mechanisms, consisting of active pulmonary vasoconstriction, which has motivated many investigators to seek novel pharmacological interventions that could mitigate the hemodynamic alterations related to this condition.^18^ In the meantime, PAH progression relies on the remodeling of smooth muscle cells and endothelial cells of the pulmonary artery, which is tightly linked to angiogenesis. The angiogenic and angiostatic factors may be potential biomarkers for PAH development.^19^ In this paper, we intended to disclose the diagnostic values of serum BNP, PTX3, and VEGF in APE‐PAH and their correlations with the severity of PAH. Collectively, we found that serum BNP, PTX3, and VEGF levels were significantly elevated in APE‐PAH patients. Serum BNP, PTX3, and VEGF levels were of clinical value in the diagnosis of APE‐PAH patients, and serum BNP, PTX3, and VEGF levels were positively correlated with the severity of PAH and could be used as predictors of the severity of PAH.

NTproBNP and BNP are produced by cardiomyocytes because of ventricular overstretch, which is regarded as the most potent blood hallmark for risk prediction of PAH.^20^ In our work, we observed higher serum levels of BNP in APE‐PAH patients and the level of which was highest in patients with severe PAP. Besides, Pearson correlation analysis signified that serum BNP levels were positively correlated with PAP grading in APE‐PAH patients, and serum BNP levels had a diagnostic power in patients. As reported, plasma BNP parameters are bound up with pulmonary hypertension and cardiac function in patients with chronic obstructive pulmonary disease (COPD) and pulmonary heart disease, implying that BNP could reflect the health condition of patients and guide their treatment.^21^ A study including 157 patients with portal hypertension or decompensated cirrhosis has disclosed that BNP levels are elevated in patients with high right ventricular systolic pressures, thus predicting portopulmonary hypertension (PoPH).^22^ Moreover, BNP measurement has been demonstrated to have utility in the early diagnosis of PoPH, with a cut‐off value of 29.1 pg/mL for BNP in the prediction of the disease.^23^

PTX3 is a novel and effective biomarker for the assessment of patients with PAH.^13^ In our work, we observed higher serum levels of PTX3 in APE‐PAH patients and the level of which was highest in patients with severe PAP. Besides, serum PTX3 levels were positively correlated with PAP grading in APE‐PAH patients, which had a diagnostic power in patients. PTX3 might be implicated in PAH pathogenesis through inflammation, endothelial dysfunction, along with cardiac functional alternations in maintenance hemodialysis patients, and PTX3 could serve as a hallmark for screening PAH.^24^ Some articles have unveiled that elevated PTX3 levels may have influences on cardiovascular events, supporting that plasma PTX3 levels could be an inflammatory marker in multiple cardiovascular clinical conditions.^25^, ^26^ Besides, elevated levels of plasma PTX3 are observed in neonates with PAH. Plasma PTX3 might be a reliable biomarker in the diagnosis of neonatal PAH.^27^

VEGF is essential for intraocular neovascular disorders and tumor angiogenesis, and VEGF plasma levels are found to be elevated in PAH patients.^15^ In our paper, we demonstrated higher serum levels of VEGF in APE‐PAH patients, and, serum VEGF levels were positively correlated with PAP grading in APE‐PAH patients, which had a diagnostic power in patients. Alkholy et al. have highlighted a notable association between elevated VEGF in PAH patients with high sensitivity and specificity, and serum VEGF levels have a positive link with pulmonary pressure.^5^ Furthermore, the cell‐based VEGF gene transfer has been demonstrated to be an effective approach for preventing PAH, which is a potential therapeutic tool in the therapy of PAH.^28^

In summary, our paper signifies that serum BNP, PTX3, and VEGF are of clinical value as biological indicators for predicting APE‐PAH and reflecting the severity of PAH. Further investigations are necessary to unveil the complex interactions of these factors in the development of APE‐PAH. However, due to the limited sample size of patients in this paper, the predictive value of the ROC curve may be difficult to apply to clinical guidance.

## AUTHOR CONTRIBUTIONS

**Bin Zhang**: Writing—original draft; Writing—review and editing. **Na Lu**: Writing—original draft; Writing—review and editing. **Xinyi Li**: Writing—original draft; Writing—review and editing. **Beibei Jin**: Writing—original draft; Writing—review and editing. **Pingkui Jin**: Writing—original draft; Writing—review and editing.

## CONFLICT OF INTEREST STATEMENT

The authors declare no conflict of interest.

## ETHICS STATEMENT

The study was implemented with the consent of all subjects and their families and ratified by the ethics committee of The First Hospital of Hebei Medical University (approval number: 20200315).

## Data Availability

The original contributions presented in the study are included in the article/Supporting Information Material, further inquiries can be directed to the corresponding author.
